# Optofluidic detection setup for multi-parametric analysis of microbiological samples in droplets

**DOI:** 10.1063/1.5139603

**Published:** 2020-04-09

**Authors:** S. Hengoju, S. Wohlfeil, A. S. Munser, S. Boehme, E. Beckert, O. Shvydkiv, M. Tovar, M. Roth, M. A. Rosenbaum

**Affiliations:** 1 Leibniz Institute for Natural Product Research and Infection Biology, Hans Knoell Institute, Beutenbergstr. 11a, 07745 Jena, Germany; 2 Faculty of Biological Sciences, Friedrich Schiller University, Fuerstengraben 1, 07743 Jena, Germany; 3 Fraunhofer Institute for Applied Optics and Precision Engineering IOF, Albert-Einstein-Str. 7, 07745 Jena, Germany

## Abstract

High-throughput microbiological experimentation using droplet microfluidics is limited due to the complexity and restricted versatility of the available detection techniques. Current detection setups are bulky, complicated, expensive, and require tedious optical alignment procedures while still mostly limited to fluorescence. In this work, we demonstrate an optofluidic detection setup for multi-parametric analyses of droplet samples by easily integrating micro-lenses and embedding optical fibers for guiding light in and out of the microfluidic chip. The optofluidic setup was validated for detection of absorbance, fluorescence, and scattered light. The developed platform was used for simultaneous detection of multiple parameters in different microbiological applications like cell density determination, growth kinetics, and antibiotic inhibition assays. Combining the high-throughput potential of droplet microfluidics with the ease, flexibility, and simplicity of optical fibers results in a powerful platform for microbiological experiments.

## I. INTRODUCTION

Droplet-based microfluidics has recently attracted significant interests from various research fields. Unique advantages such as miniaturization, lower reagent consumption, faster sample processing (at kHz or higher), flexibility in manipulation (splitting, merging, etc.), and low cost have made it the new paradigm for high-throughput experimentations.^1–5^ It enables rapid analysis of single cells, biomolecules, and chemicals from libraries consisting of millions of variants. In the last 15 years, droplet-based microfluidics really took off in various biological and biochemical applications including enzyme screening,^6–8^ single cell studies,^9–11^ screening of antibiotic-resistant mutants,^12^ directed evolution,^13,14^ and nucleic acid analysis.^15^ In microbiology, droplet microfluidics has opened new possibilities to detect and identify microorganisms, and study microbial physiology and biotechnological applications.^4^

Microfluidic systems have enabled miniaturization that achieves the handling of picoliter volumes. Despite the effort in miniaturizing the fluid handling operations, there has been very limited development in the detection and analysis of contents. Traditional microfluidic detection setups depend on conventional free-space optics with arrays of lenses and dichroic mirrors along with fluorescence microscopes.^16–20^ The complex integration and alignment of these free-space components render the entire system bulky and expensive.^21,22^ The rigidly fixed components leave only little freedom for flexibility in design of experiments and require tedious handling and maintenance. The complex and rigid optical detection systems in general create a high-entry barrier for traditional biological researchers to this technique.

With recent developments in incorporating miniaturized optical components into microfluidic chips,^23,24^ optofluidics can provide solutions to bulky, rigid, and complex free-space optics. Optofluidics is defined as a synergistic integration of optical components into a microfluidic system.^25^ Different optofluidic projects have been designed and manufactured by integrating micro-optical components,^26^ such as air lenses,^21,24^ waveguides,^27,28^ fibers,^21,29^ fluidic lenses,^30^ prisms,^31^ and mirrors,^24,32^ In particular, optical fibers have been integrated into microfluidic chips as light guides and as fiber-based sensors,^33^ where in the latter case optical fibers are modified to become a sensing device. Optical fibers, used as light guides, function similar to waveguides as a light transmission medium. Miniaturized waveguides have been demonstrated in microfluidic systems,^34,35^ but the on-chip waveguides are generally complex, expensive, and time-consuming to fabricate. Besides, waveguides suffer from high transmission losses (in the range of dB/cm) in comparison to conventional optical fibers (in the range of dB/km). Optical fibers are flexible, low-cost, easy to integrate, and compatible with a wide range of wavelengths.^3,36^ These superior features make optical fibers potentially interesting for diverse microfluidic applications.

Optical fibers have been embedded for measuring and quantifying different analytes in droplet microfluidics. In most of the optofluidics studies, optical fibers are basically integrated for obtaining fluorescence,^3,9,37,38^ scattered,^12,39^ or absorbance signals.^13,22,40^ So far, these studies are focused on single detection techniques and lack the capability of multiplexing several detection signals. Simultaneous detection of multiple parameters empower many chemical and biological experiments since it gives a higher information density from which researchers can deduce better conclusions and control the experimental processes more precisely.^29,41–43^ Thus, here we focus on the development of a multi-parametric detection setup and simultaneous data collection. With multiple optical fibers integrated into a microfluidic chip and coupled to state of the art light sources and detectors, the application range of a microfluidic setup can be expanded from simple analytical assays with measurements of only a single parameter to complex multi-parametric chemical/biochemical experimentation with high content read outs. Furthermore, fibers were previously placed on the chip subjectively rather than according to the actual beam characteristics of optical fibers.^21^ We suggest instead the use of micro-lenses for beam focusing and collimation in combination with fiber integration in order to improve the signal to noise ratio. Hence, for the development of a refined multi-parametric detection setup, a microfluidic chip with integrated optical fibers and properly designed micro-optical components is required.

**FIG. 1.**
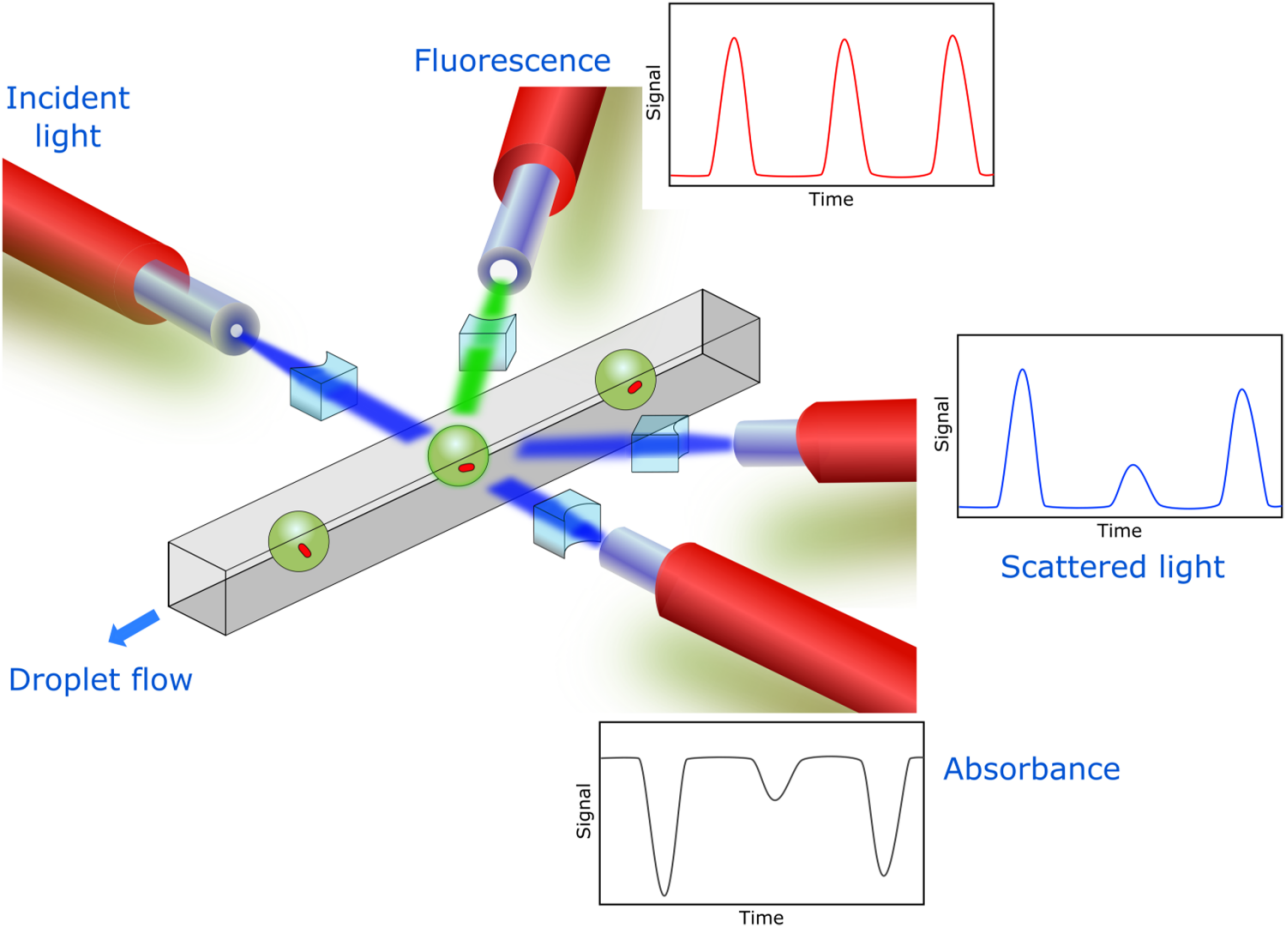
Scheme of an optofluidic platform for simultaneous multi-parametric detection. Multiple fibers are embedded into a microfluidic chip for detection of different parameters (absorbance, fluorescence, and scattered light).

This research describes the design, fabrication, characterization, and application of a flexible, simple, and cost-effective optofluidics platform with integrated optical fibers, micro-optical components, and multiplexed optical detection (Fig. 1). Fluidic micro-channels, guide structures for inserting optical fibers, and micro-lenses were micro-fabricated on a polydimethylsiloxane (PDMS) chip. Embedded optical fibers successfully guide incident light and collect transmitted, emitted, or scattered light toward the detectors. The developed optofluidic platform was initially validated for measurement of absorbance, fluorescence, and scattered light and ultimately utilized for various microbiological applications including growth kinetics and antibiotic inhibition studies. This work demonstrates the flexibility and capability of optical fibers embedded in a microfluidic setup for the simultaneous detection of multiple parameters and their application for microbiological experimentation.

## II. MATERIALS AND METHODS

### A. Chip fabrication

Microfluidic chips were fabricated by using soft lithography in PDMS from glass molds produced with femtosecond laser machining technology as previously described.^44^ Briefly, fluidic and optical parts were designed in AutoCAD 2015 (Autodesk Corp, USA). Molds were fabricated on fused silica slides with Femtoprint technology (FEMTOprint SA, Switzerland). Intermediate PDMS molds and working chips were produced by PDMS replica molding. Standard PDMS (Sylgard 184, Dow Corning) mixture (10:1 mixture of base and curing agent) was poured onto the mold, degassed, and thermally polymerized at 70 °C for 3 h. The polymerized PDMS was peeled off from the mold and cut into individual chips. Inlet and outlet ports were punched in the PDMS using micropunches for fluidic connections. Finally, PDMS chips were plasma bonded (Zepto, Diener) to a microscope glass slide. All fluidic channels were treated with Trichloro(1H,1H,2H,2H-perfluorooctyl)silane (Sigma).

### B. Optical fiber integration and optical configuration

The tips of all optical fibers were stripped off of their outer protective and jacket layer with only the core and cladding layer remaining. Stripped fibers were cleaved with a fiber scribe to obtain a flat surface at the fiber tip end. The flatness at the fiber ends was confirmed by microscopic imaging. Optical fibers were inserted manually into the chip through the respective fiber guide structures with careful observation under a stereo microscope and fixed with instant glue to the glass slide. The air gap between the fiber tip and microchannels was removed by injecting uncured PDMS through specific inlets and letting it cure at room temperature overnight. The excitation fiber (fiber patch cord, single mode, polarization maintaining, LASOS) has a cladding diameter of 125 *μ*m and a core diameter of 6 *μ*m with a numerical aperture (NA) of 0.14. It is connected to a beam combiner consisting of lasers with different wavelengths (488 nm, 561 nm, and 639 nm). Detection fibers (FC fiber patch cable, multimode, Thorlabs) have a cladding diameter of 125 *μ*m and a core diameter of 50 *μ*m with an NA of 0.22. For transmitted light detection, light collected by the detection fiber is coupled to a photodiode with a bandpass filter (470/40 nm) and a neutral density filter (ND2) stacked together. Fluorescence light collected by a detection fiber is directed to a photomultiplier tube (PMT) with a bandpass filter (525/10 nm). Similarly, for the scattered signal, the intensity of light from the detection fibers is measured by a PMT with a quad-bandpass filter (440/521/607/700 HC Quadband Filter, AHF).

### C. Microfluidic actuation and imaging

A high-precision syringe pump (neMESYS, CETONI) was used for fluid control. Polytetrafluoroethylene (PTFE) tubings were inserted into the inlets and outlets of the microfluidic chip. A high-speed camera (acA1920-150uc, Basler) was used for monitoring the microfluidic operations on a stereo microscope (Stemi 2000, Carl Zeiss). Imaging of stationary droplets was performed with a PCO.edge 5.5 m camera (PCO) and a Spectra-X Light Engine (Lumencor) using an inverted microscope (Axio Observer Z1, Carl Zeiss).

### D. Microorganisms and culture conditions

Culture media TB and MMM were prepared as described previously.^45^ *Escherichia coli* ECJW992, which is producing the red fluorescent protein mCherry after IPTG induction,^45^ was cultured overnight in TB media with 10 mg/l glucose and 100 *μ*g/ml ampicillin. From the overnight culture, a pre-culture was inoculated to defined optical density at 600 nm (OD) of 0.1 and grown until mid-exponential phase. For droplet generation, the pre-culture inoculum was diluted to the required optical density using a fresh TB medium along with 0.5 mM IPTG. Spores of the mycelia-forming bacterium *Streptomyces hygroscopicus* were added to the MMM medium to a final concentration of 5 × 10^7^ spores/ml and used for droplet generation. Generated droplets were collected and incubated in the dynamic droplet incubation setup^45^ placed in a humid chamber at 28 °C.

### E. Chemicals and reagents

Carboxyfluorescein, 3-bromobenzotrifluoride, and p-nitrophenol were purchased from Sigma. Carboxyfluorescein and p-nitrophenol were diluted using a phosphate-buffered saline buffer into different concentrations. Green food dye (Wusitta, Erich Wutzig) was diluted using de-ionized water to form a series of dye solution with concentrations from 100% to 0%. In growth inhibition assays, carboxyfluorescein and far-red dye (DY-654, Dyomics) were applied to label droplets. Novec (HFE7500, 3M) oil with 0.5% Picosurf 1 (Dolomite) was used as a continuous phase for droplet generation.

## III. RESULTS AND DISCUSSION

### A. Design and characterization of an optofluidic chip

**FIG. 2.**
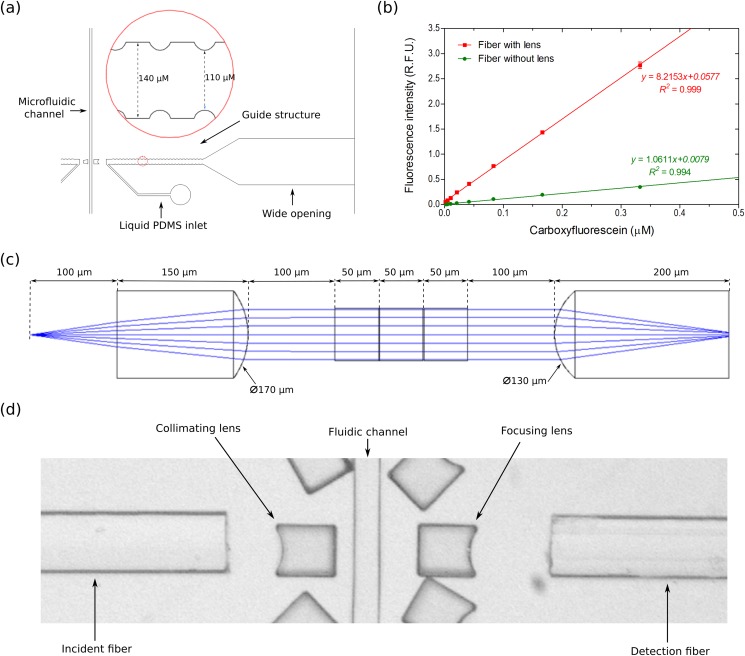
Design and characterization of the optofluidic chip. (a) AutoCAD design of the fiber guide structure. (b) Calibration curves for carboxyfluorescein using chips with integrated micro-lenses and without lenses. Each dataset consists of ∼1300 droplet measurements. Error bars represent 1 standard deviation. (c) Zemax simulation of a light beam from the fiber being collimated and focused through micro-lenses. (d) Microscopic image of the optical interrogation region in the microfluidic chip with integrated optical fibers.

Optical fibers improve the flexibility and minimize the complexity of microfluidic detection setups. For easily integrating optical fibers, a microfluidic chip was designed with specialized structures including fiber guide channels and micro-lenses. First, a guide channel with inward and outward grooves as previously mentioned^24^ was designed in a PDMS chip to align and clamp the fiber tightly. The guide structures were designed and optimized for standard optical fibers of 125 *μ*m cladding diameter, so that optical fibers could be efficiently clamped. The distances between two opposite inward and outward grooves were 110 *μ*m and 140 *μ*m, respectively [Fig. 2(a)]. This distance can be easily changed during chip-designing in relation to the dimension of the fibers.

A wide opening followed by a gradual decrease to a narrow guide structure was designed at the end of guide structures for an easy insertion of fibers [Fig. 2(a)]. The wide opening is 5 mm wide and is visible for the human eye. Thereby, an easy initial insertion is possible, bringing the fiber to the right position in the guide structure. Thus, fiber insertion can be done manually without requiring specialized optical tools. Inlets adjacent to the fiber guide structures were also included in the design for allowing the injection of liquid PDMS to fill the air gap between the fiber end tip and the PDMS wall of the guide structure.

Due to the nature of light transmission through an optical fiber, the light emerging at the end of a fiber is divergent. For many chemical and biological analyses, uniform and even light distribution is desired for sample excitation.^46^ Therefore, a micro-lens was designed between the fiber and the micro-channel for focusing and collimating the light beam as previously mentioned,^24,37,47^ thereby enhancing the system performance. The difference of refractive indices from the microfluidic chip material (PDMS) and air is utilized for designing PDMS-air micro-lenses. These lenses can be easily integrated and fabricated by single-step soft lithography as they only need to be included in the initial design. These lenses do not require any further modifications or adjustments after production of the chip.

A ray tracing simulation was conducted in Zemax to design a micro-lens that can collimate light with fixed beam diameter [Fig. 2(c)]. During the simulation, the beam diameter at the center of the channel was set as a merit function (50 *μ*m). Other parameters such as the radius of the lens curvature, the distance between fiber end and lens, the distance between lens and fluidic channel, and the length of the lens were set as variables. The refractive index of PDMS (prepared at 10:1 ratio of polymer and cross linker) is considered as 1.4.^35,48^ Based on the simulation, the optimized curvature diameters for a concave lens for light collimation and for focusing transmitted light were determined to be 170 *μ*m and 130 *μ*m, respectively. The final lens design with dimensions and a microscopic photograph of a fabricated lens is shown in Figs. 2(c) and 2(d).

The PDMS chip with fluidic micro-channels, fiber guides, and micro-lenses was characterized with a Laser Scanning Microscope (LSM) in order to confirm and determine proper dimensions and local roughness. The fluidic channel width and height divergence were measured 2 *μ*m and 1 *μ*m in average, respectively (Fig. S1 in the supplementary material). Since fibers are embedded from the side, perpendicular to the microfluidic channel, smooth and flat surfaces are desired in the vertical section of the microfluidic channels and micro-lenses to reduce light attenuation and transmission losses.^49,50^ Determined by LSM measurement, the typical average roughness (Ra values) was 150 nm on the vertical sections (Fig. S2 in the supplementary material). Even though the Ra value is slightly higher than in chips fabricated from SU8 molds, it is better than other 3D printed chips.^51^

To characterize the performance and importance of integrated micro-lenses, the fluorescence signal of carboxyfluorescein was compared between chips with integrated lenses and chips without lenses. While using the same concentration of carboxyfluorescein in the aqueous phase and same laser intensity, the fluorescence signal intensity was about eight times higher with integrated micro-lenses [Fig. 2(b)]. This result confirms the beam path collimation and focusing of emitted light by micro-lenses. The lenses are important for maximizing the amount of excitation light and emitted light collected with the detection fiber by focusing the light beam to a fixed diameter, which should be equal to the core diameter of detection fiber.

Furthermore in our setup, considering an optical fiber with a core of 50 *μ*m and cladding of 125 *μ*m, a multi-level chip was designed for increasing signal intensity and detection sensitivity.^44^ The multi-level chip allows the axis of the optical fiber to be always at the same height as the center of microfluidic droplets in the channel. Thereby, the droplets are in the center of the beam path and get fully excited leading to a higher signal intensity and improved sensitivity. Chip layers of 125 *μ*m depth for fiber guides and 87.5 *μ*m for fluidic channels were fabricated. Insertion of 50 *μ*m core diameter fiber results in a beam path centrally aligned to droplets in the fluidic channel.

### B. Multi-parametric measurements

**FIG. 3.**
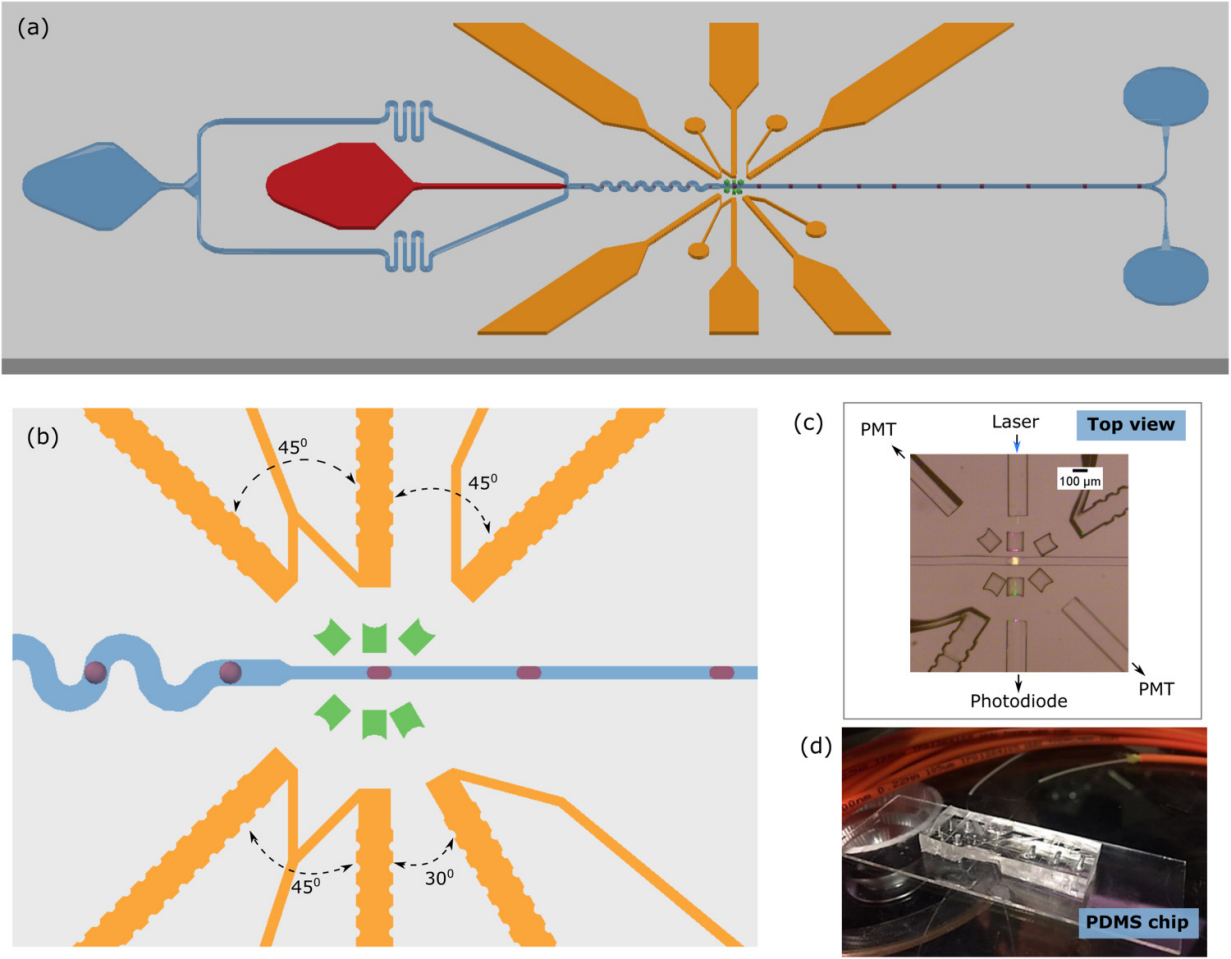
Optofluidics chip for multi-parametric measurement. (a) Schematics of a droplet formation chip with fiber guide structures (yellow), micro-lenses (green), channels filled with oil (blue), and aqueous phase (red). (b) Optical interrogation region showing fiber insertion angles. Absorbance, fluorescence, and scattered lights were measured with fibers inserted at different angles. (c) Microscopic image of the interrogation region on a chip. (d) Photograph of fibers integrated in a PDMS chip in the lab.

With the introduced features, an optofluidic platform was established that exploits the facile integration of multiple fibers for simultaneous detection of various parameters. The optical fiber arrangement was designed to guide the incident light into a sample at an optical interrogation zone by a single input fiber. The simultaneous detection of signals was realized by multiple fibers positioned at different angles (Fig. 3). During chip designing, the fiber guide structures were carefully placed in such a way that the central axis of all fibers would intersect at the optical interrogation point [Figs. 3(b) and 3(c)]. An excitation fiber was embedded perpendicular to the fluidic channel. Three detection fibers were used for measuring signals for transmitted, scattered, and fluorescence light. For transmission, a detection fiber positioned orthogonally to the excitation fiber was used. Fibers inserted at multiple angles (45°, 135°, and 225° to excitation fiber) were used for measuring fluorescence and scattered light.

At the droplet interrogation region, the width of the fluidic channel is constricted from 100 *μ*m to 50 *μ*m for squeezing droplets to minimize the curved surfaces and prevent light reflections. The central part of the squeezed droplet completely touches both sidewalls of the microfluidic channel [Fig. 3(b)]. Besides, this reduces the oil layer in the fluidic channel, which decreases light loss due to light reflection at the same time.

The developed optofluidics setup was initially optimized for three different parameters separately, as described in Secs. III B 1–III B 3, and was demonstrated for different microbiological applications.

#### 1. Absorbance measurement

Absorbance is a label free detection technique for quantification of an analyte concentration. It is a basis for a large number of conventional enzymatic assays. Unfortunately, absorbance measurement in microfluidics is hindered due to the miniaturization of fluidic channels leading to decreased optical path lengths by typically two to three orders of magnitude.^28,40,52^ Therefore, fluorescence-based detection assays are much more common in droplet microfluidics while absorbance-based assays are rarely adapted for droplets. In the developed setup, absorbance is successfully measured by using two properly aligned optical fibers positioned orthogonally to the microfluidic channel along with micro-lenses, which allow optimized light beam delivery and reduced background noise.

A typical transmitted light pattern of droplets is observed in Fig. S3 in the supplementary material. It presents characteristic leading and lagging spikes with a plateau between them. Spikes arise due to the difference in refractive indices of the oil phase and the droplet aqueous phase, causing regions of total internal reflections that are seen as the borders of the droplet. By changing the refractive index of the oil phase, the spike levels can be diminished. Various chemicals with oil solubility have been reported as refractive index modifiers^53^ of perfluorinated oil, such as 1,3-bis(trifluoromethyl)-5-bromobenzene, 3-bromobenzotrifluoride and 3-iodobenzotrifluoride among others. We tested 25% of 3-bromobenzotrifluoride in the oil phase, which resulted in lower spike levels during absorbance measurements for droplets (Fig. S3 in the supplementary material). With the removal of spikes, the transmitted light signal becomes easier and more reproducible to analyze.

**FIG. 4.**
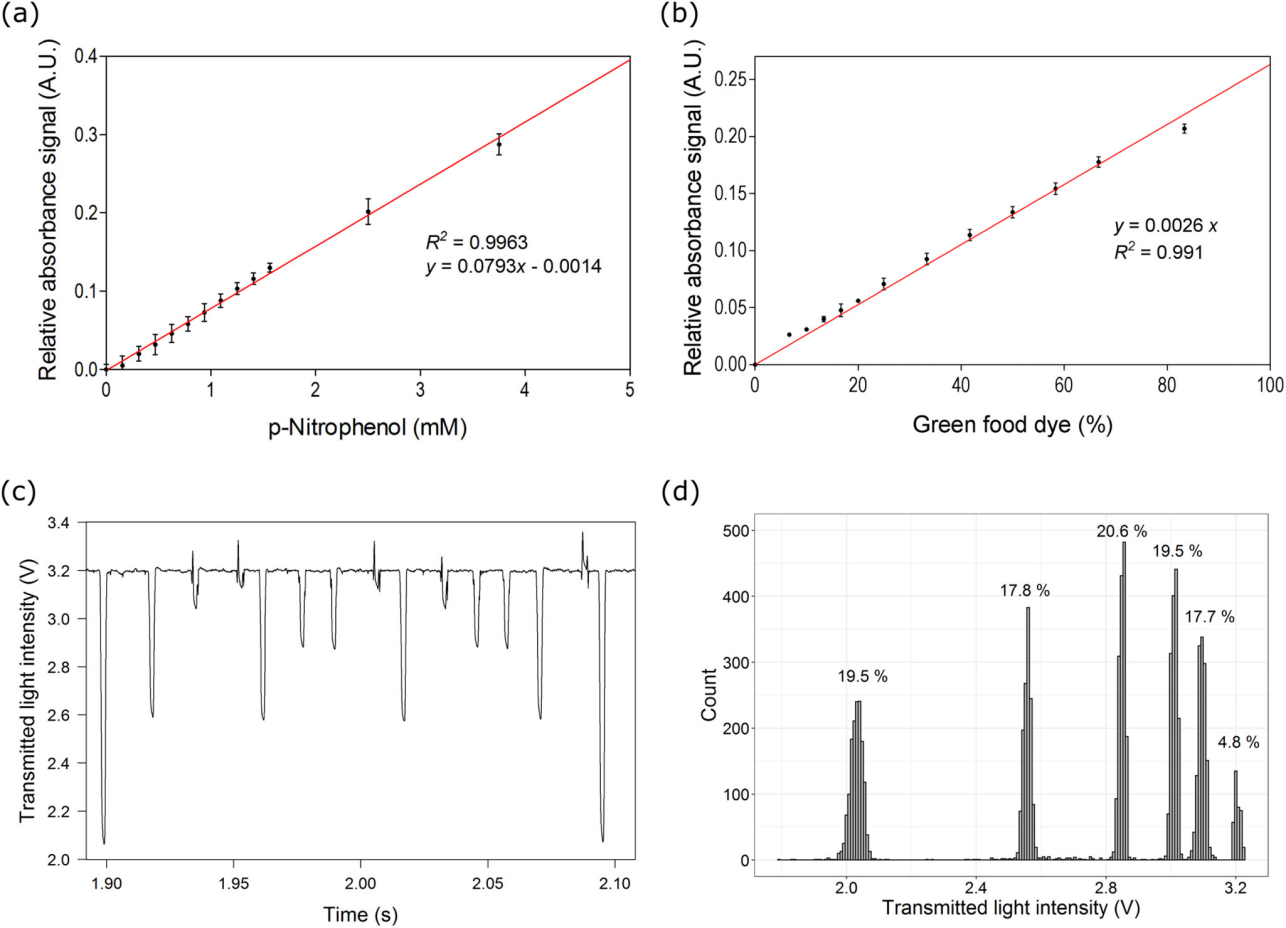
Absorbance measurement using the optofluidics platform. Calibration plot for (a) p-nitrophenol and (b) green food dye. A minimum of 10 000 droplets were analyzed for each concentration. (c) Transmitted signal pattern for reinjected droplets from the mixed droplet population containing six different concentrations of green food dye. (d) Histogram showing the distribution of transmitted light intensities with six distinct clusters corresponding to the six different droplet subpopulations. Error bars in (a) and (b) indicate 1 standard deviation.

To demonstrate the absorbance measurement, transmitted light from droplets with different concentrations of p-nitrophenol and green food dye were measured. Figures 4(a) and 4(b) show that the absorbance signal increases linearly with the concentration of dye with a detection limit (LoD) of 0.25 mM for p-nitrophenol and 0.21% for green food dye (corresponding to three standard deviation of the blank sample). The obtained LoD is acceptable considering the fact of limited effective path length and is sufficient to extract quantitative information in various biological applications including microbial biomass and chromogenic assays. The sensitivity of the absorbance measurement at lower dye concentrations could be further improved by modifying the channel geometry for extending the light path length,^28,52^ creating reflecting mirrors,^32^ and using differential photothermal interferometry.^54^

The applicability of this approach was validated through the analysis of a diverse population of droplets, in which six different dye concentrations (100%, 50%, 25%, 12.5%, 6.25%, and 0%, diluted in water) were generated sequentially and mixed in equal proportion. The mixed droplet population was reinjected (∼100 droplets/s) into the microfluidic chip, and the transmitted light signal per droplet was measured. An exemplary droplet signal pattern is shown in Fig. 4(c). Based on the absorbance signals, the mixed droplets can be differentiated into six distinct sub-populations [Fig. 4(d)]. The detected frequencies for all sub-populations were similar (17%–20%) except for one (4.8%). This is due to the fact that this subpopulation had transmitted signals above baseline and was not properly detected during data analysis. However, all six subpopulations were separable to distinct histogram peaks. All together this implies that absorbance can be used for quantitative measurements, distinguishing droplets by their absorbance properties.

#### 2. Scattered light measurement

**FIG. 5.**
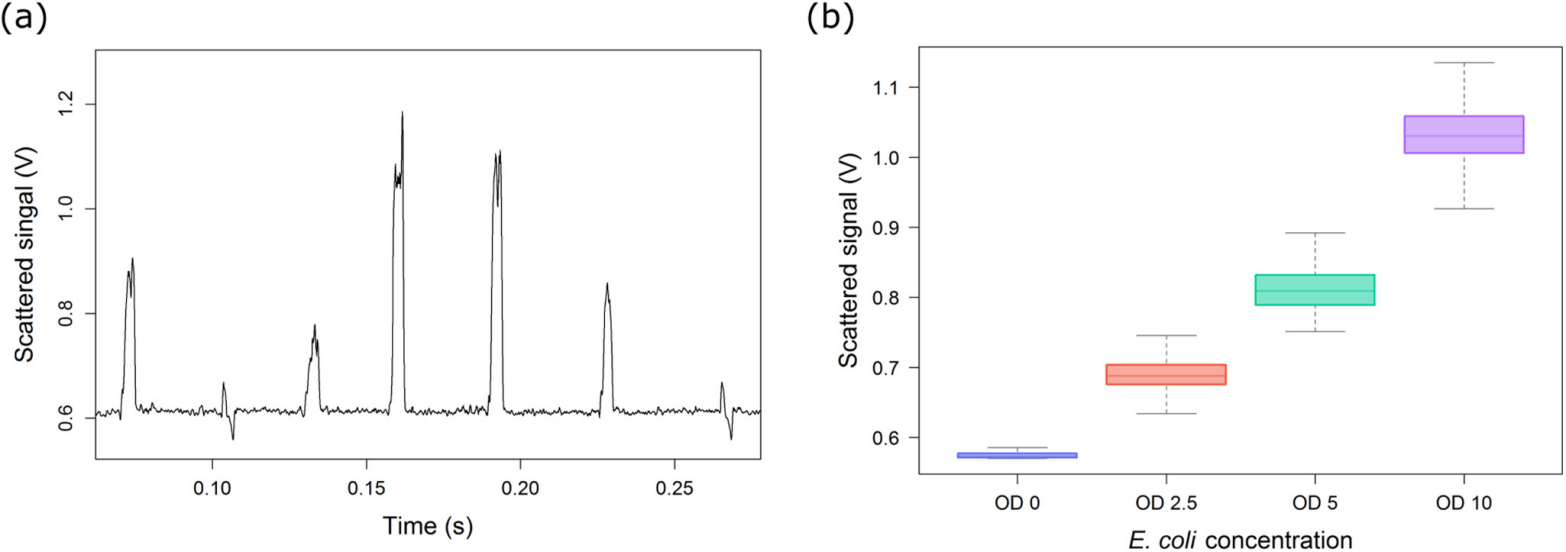
Scattered light measurement with optical fibers. (a) Typical scattered signal pattern from droplets with different cell densities. (b) Box plot showing dependency of scattered signal with cell concentration. Error bars indicate 1 standard deviation.

We measured the scattered light intensity in certain angles from droplets containing *E. coli* cells at various known cell densities. The dependency between the microbial cell concentration of droplets and the detected scattered signal is displayed in Fig. 5. Droplets with different cell densities (OD 10, 5, 2.5, and 0) were generated, mixed, and reinjected. Typical scatter signals can be seen in Fig. 5(a). Scattered signal peaks with different intensities resemble the droplets with different cell concentrations. An increase in cell concentration showed an increment in the scattered light [Fig. 5(b)]. At higher concentrations, variability in the measured signal increased. This might be due to several factors like growth variability in individual droplets and cell distribution inside droplets. The measured scattered values provide information on cell density in droplets and could be used for differentiating microbial growth phases. Each box in the figure was obtained from hundreds of uniform droplets at each cell density. This demonstrates that our setup is able to detect scattered light and differentiate droplets with different cell densities.

The scattered light intensity at different angles provides additional important information. Forward scattering measured at low angles (between 0° and 5° along the excitation light path) shows variation in shape and size, while side scattering measured at larger angles depends on granularity of the sample. Both parameters are required for identifying cells or particles.^55^ Light scattering measurements at different angles are possible by inserting multiple fibers at different angles as they are very small and can be oriented at the center of the fluidic channel. A single excitation fiber could be coupled to multiple detection fibers for obtaining angular light scattering patterns, which potentially could be used for detecting different types of microorganisms in droplets.^56^

#### 3. Fluorescence measurement

**FIG. 6.**
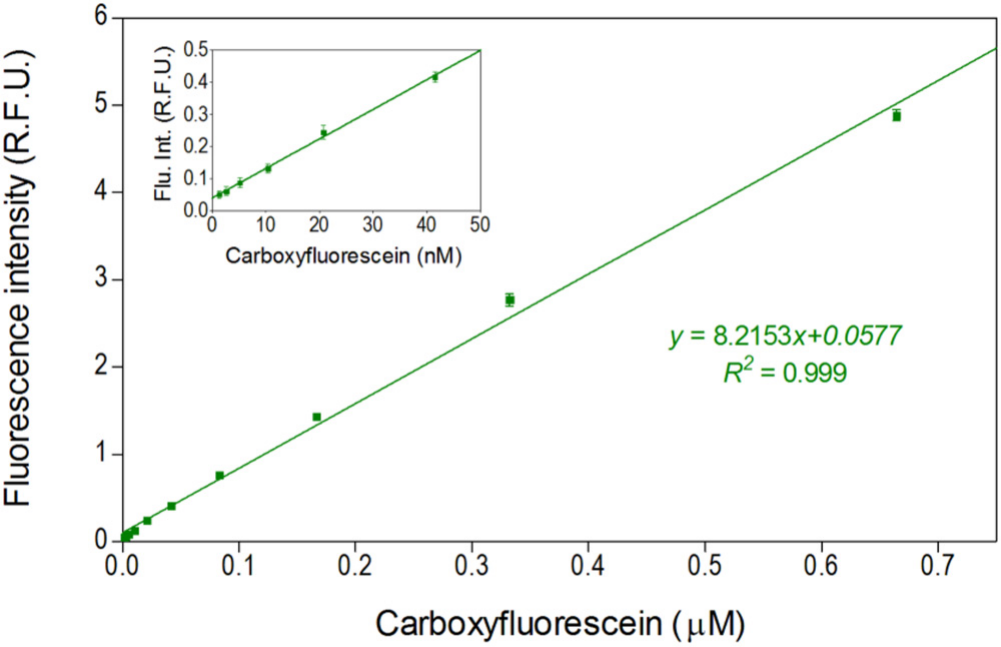
Calibration plot for carboxyfluorescein. Inset picture shows a calibration plot at lower concentration. A minimum of 2700 droplets were analyzed per each concentration. Error bars indicate 1 standard deviation.

The developed setup was investigated for fluorescence detection efficiency. Carboxyfluorescein was used as a reference dye for characterization of the fluorescence measurement. An excitation fiber is coupled to a laser and integrated into a microfluidic chip. The fluorescence signal emitted by fluorescent dyes is collected by an emission fiber and detected by a photomultiplier tube (PMT). Droplets of different concentrations of carboxyfluorescein were generated and the fluorescence intensities were measured. An increase in the dye concentration resulted in a linear increase of fluorescence intensity with a *R*^2^ value of 0.99 (Fig. 6). The limit of detection, defined as the concentration, which gives a signal equal to three times the standard deviation of the blank, for this optofluidic setup was determined to be 2 nM. The dynamic range for the detection spanned from 2 nM to 0.7 *μ*M. These results prove compatibility of the developed setup for high-efficient fluorescence measurements in various analytical and biochemical applications. Furthermore, multiple fibers can be inserted at different positions^38^ or combined with other techniques like frequency division multiplexing^57^ for detecting multiple fluorescence colors and could be used for different assays where detection of multiple colors are required.^11^

### C. Microbiological applications

**FIG. 7.**
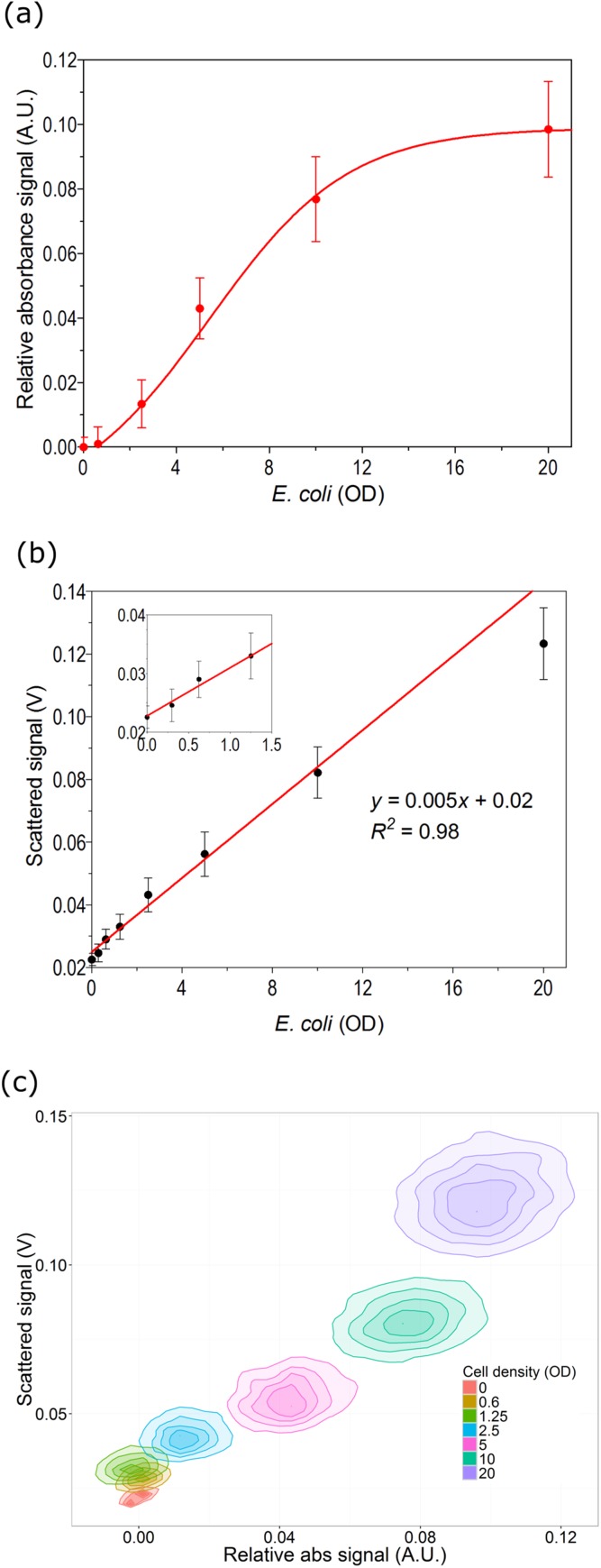
Measurement of bacterial cell density with the optofluidic platform. (a) Dependency of the absorbance signal on cell density. Absorbance signal increases with the increase in cell density in droplets. (b) Dependency of the scattered light signal on cell density for *E. coli*. (c) Discrimination of cell populations with different cell densities by using measured values for absorbance and scattered light of each droplet. Error bars indicate 1 standard deviation.

Building up on the characterization of various read out techniques of the optofluidic setup, combinations of the single techniques were tested on droplets containing microbial samples. Droplets with defined cell densities of *E. coli* were generated. Absorbance and scattered signals were simultaneously measured for each droplet in order to plot a calibration curve. As expected, with an increase in cell concentration, we observe an increase in absorbance and scattered signals (Fig. 7). For the absorbance signal, the obtained dilution plot shows linear response in an OD range of 2–10. At cell densities below 1.5, the measured absorbance signals were barely distinguishable from blank droplets. This could be due to the limited sensitivity at a very short light path length due to the size of the droplet. Yet our setup is applicable to many biological applications requiring differentiation of grown cells from non-grown or less grown cells. However, the detection sensitivity at lower cell densities can be improved by staining cells or using cellular activity-based colorimetric dyes.

Increment of a scattered light signal was observed depending on cell concentration, and linear fitting resulted in a *R*^2^ value of 0.98 [Fig. 7(b)]. Although there is higher variability at lower cell densities (below 1.5), the plot shows a linear response in an OD range of 0.5–12. This indicates that our setup is capable of quantitatively analyzing the concentration of cells in droplets. The measured two parameters were utilized for differentiating droplets of different cell densities [Fig. 7(c)]. Distinctly separable clusters were observed for some cell densities, which were barely distinguishable from single parameter analysis. Scattered signals of droplets with OD 5 and OD 2.5 were overlapping and were difficult to differentiate. While in combination with absorbance signals, these two populations were classified into two separable clouds. Thus, by combination of two parameters, we could differentiate/classify droplets with various cell concentrations.

**FIG. 8.**
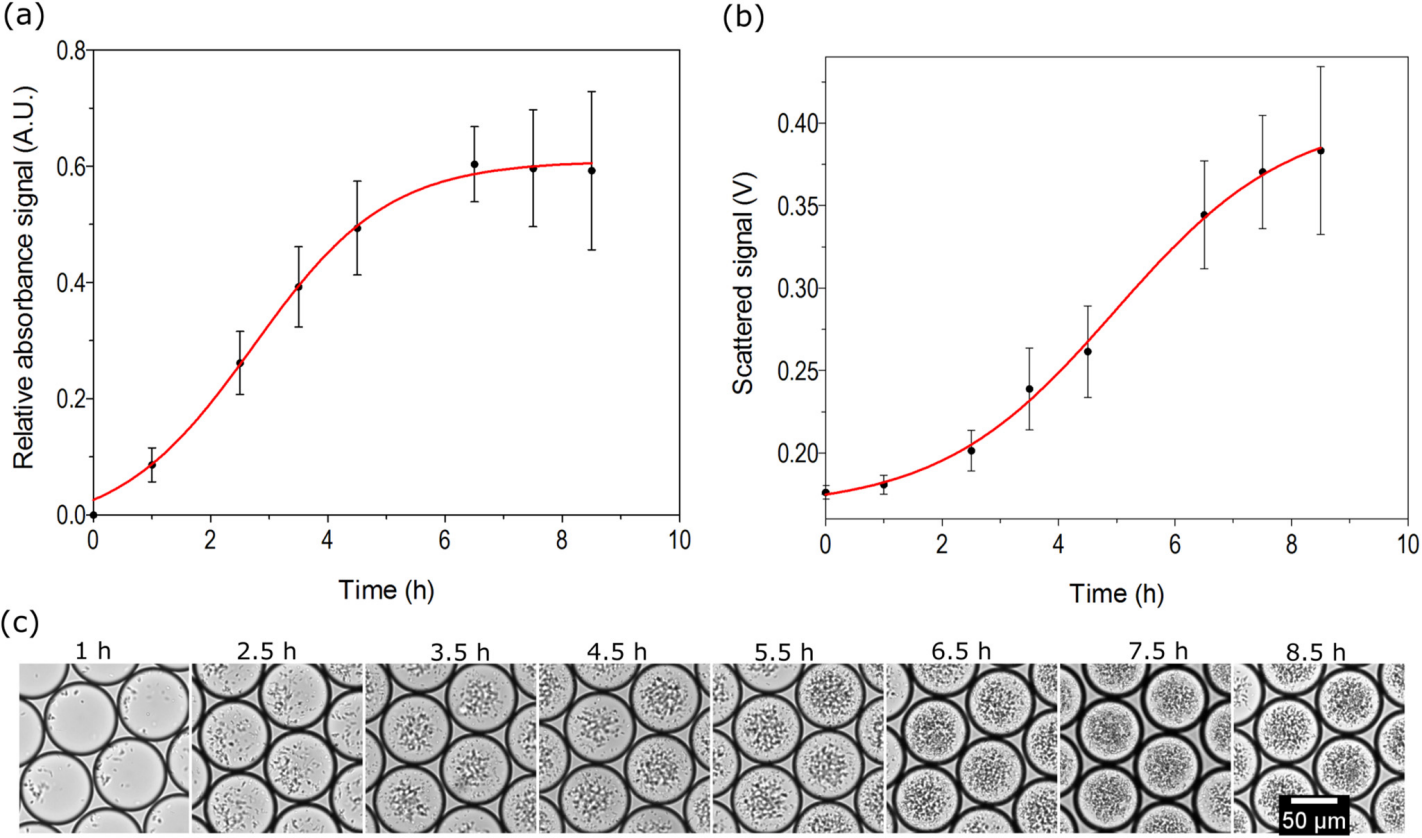
Investigation of bacterial growth kinetics in droplets. Growth of *E. coli* was measured at different time points in droplets by using two parameters simultaneously: (a) absorbance and (b) scattered light. (c) Microscopic images of droplets at different time points. Around 600 droplets were analyzed for each measurement point. Error bars represent 1 standard deviation.

Droplets are providing a high-throughput alternative to shake flasks and micro-titer plates for microbial culture.^45,58^ Following growth kinetics is important for understanding different stages of microbial growth as it is the basis for different biological and biotechnological applications. Here, we used our setup for quantitatively measuring growth kinetics of *E. coli* and the mycelia-forming bacterium *S. hygroscopicus*. *E. coli* cells were encapsulated in droplets, starting with 50 cells/droplet. Generated droplets were incubated at 28 °C using our previously established system for dynamic droplet incubation.^45^ Absorbance and scattered light were measured at various incubation time points using the developed setup. Figure 8 shows the growth curves of *E. coli* obtained from two different detection parameters. It was observed that *E. coli* cells reached the stationary growth phase about 7 h after incubation. For *S. hygroscopicus*, it took 45 h to reach the stationary growth phase (Fig. S4 in the supplementary material). Images of the droplets confirm the results. We observed a good agreement between image data and measured data (Fig. S5 in the supplementary material).

Higher variability of measured values was observed with time in both detection methods when the microbial cultures in the droplets were reaching the stationary phase. This can be attributed to growth variability in individual droplets due to cell-to-cell heterogeneity and phenotypic diversity of microbial cells.^59^ Naturally, in biological replicate growth experiments with a very small inoculum cell number, individual variability in cell fitness will cause larger droplet population heterogeneity than typically observed for parallel larger scale cultivations. While these variabilities are undetermined and averaged out in traditional shake flask cultures, droplet microfluidics can be implemented for studying phenotypic diversity and provide possibility for isolating strains with differing phenotypic properties.

**FIG. 9.**
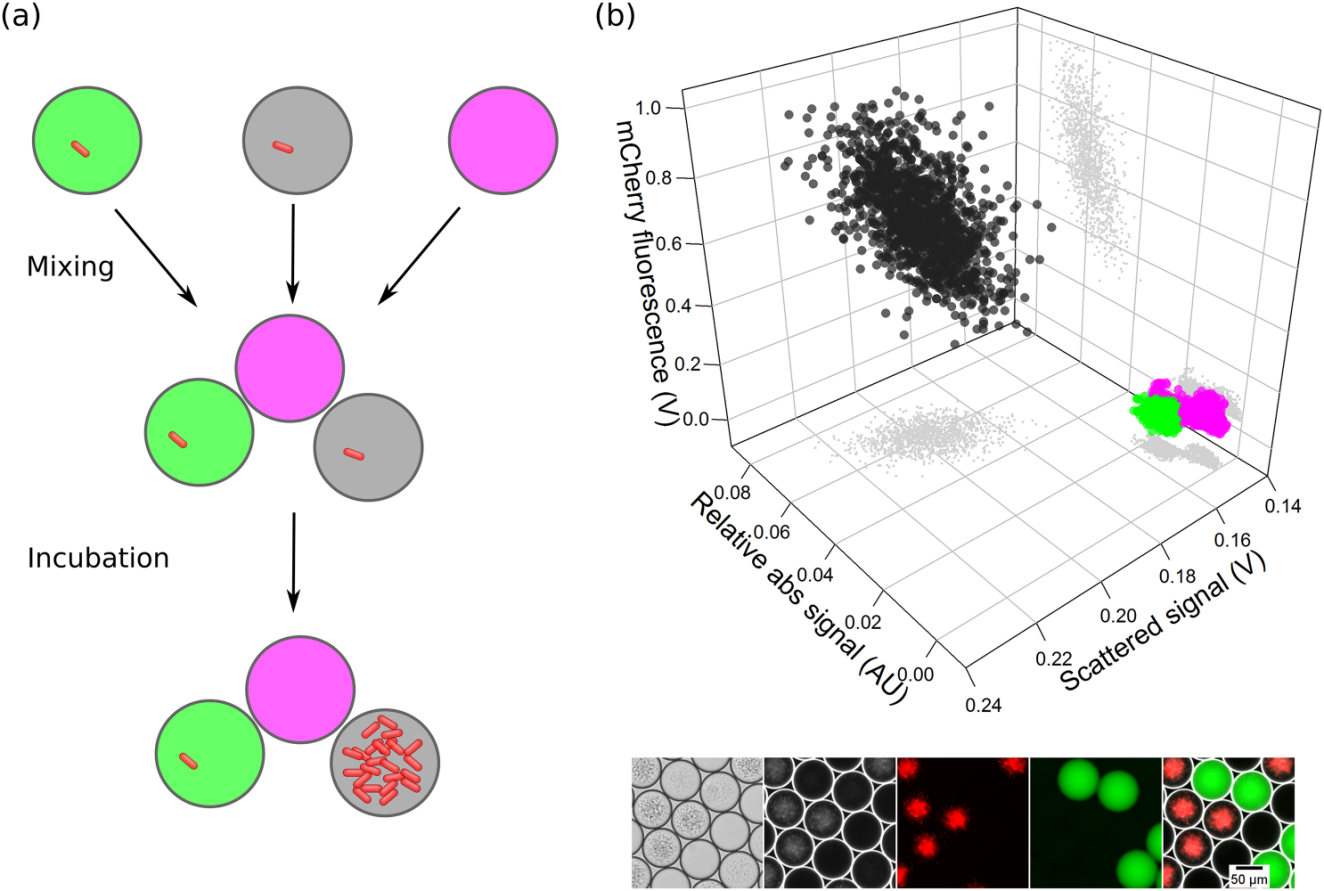
Microbial inhibition analysis for antibiotics. (a) Schematic of a model inhibition assay. In droplets containing an antibiotic (green labeled) bacterial growth is inhibited, whereas in droplets without antibiotic (unlabeled, gray) bacterial cells proliferate. Empty droplets (far-red labeled) are used as control droplets. (b) The three populations were classified into three clusters by simultaneously measuring absorbance, scattered, and fluorescence signals. Droplets with antibiotic and inhibited cell growth had low red fluorescence and scattered light signal (green cluster). Droplets without antibiotics and cell growth had high absorbance and red fluorescence signal (black cluster). Small gray dots represent 2D scatter plots of measured signals. Lower images are microscopic images of droplets in brightfield, darkfield, red, green, and overlay of fluorescence channels. Far-red fluorescence is not visible in microscopic images.

Growth inhibition assays are highly important for testing the activity of antibiotics in different microorganisms. Traditional assays performed in agar plates can now be performed at much higher throughput in droplet microfluidics.^60^ We designed our inhibition assay to differentiate non-inhibited bacterial cells from inhibited bacterial cells in the presence of an antibiotic. The growth of susceptible *E. coli* cells producing the red fluorescent protein mCherry is suppressed in the presence of the antibiotic tetracycline, whereas cells inside droplets without an antibiotic continue to grow and replicate many times. Thus, droplets with inhibited cells can be differentiated by lower cell density and a lower red fluorescence signal. Figure 9(a) shows the schematics of the performed inhibition assay. To demonstrate this approach in our optofluidics setup, three droplet populations were generated, (i) droplets containing bacterial cells with inhibiting antibiotic and a green fluorescent label, (ii) droplets containing bacterial cells without inhibiting antibiotic (unlabeled, red fluorescence of mCherry expected), and (iii) droplets of a culture medium with a far-red fluorescent label but without bacterial cells. These three droplet populations were mixed and incubated for 24 h to allow cells to proliferate. Droplets were reinjected into the chip and multiple parameters (absorbance, scattered, and fluorescence signal) were measured simultaneously using the developed optofluidic setup.

Inhibition of *E. coli* can be seen as expected in the subpopulation containing tetracycline, whereas no inhibition was observed in the subpopulation without antibiotic. We could see an increase in scattered light signal and fluorescence intensity in relation to growth of *E. coli*. Empty droplets were included as a control population where no bacteria were present and thus had low scattered signal and fluorescence. This result validates the successful implementation of an inhibition assay using our developed setup. The developed inhibition assay with optofluidic setup could be implemented in various applications such as screening natural soil samples for microorganisms producing bioactive compounds, mutant library screening for improved activity, and testing for activation of silent biosynthetic gene clusters responsible for antibiotic production. Furthermore, it could be implemented for microbial toxicity testing against different chemicals and determining minimum inhibitory concentration levels.

## IV. CONCLUSION

Herein, we have demonstrated an optofluidic platform with integrated optical fibers and PDMS-air micro-lenses for establishing a multi-parametric measurement system. Multiple fibers were embedded at different angles for measuring various parameters simultaneously. Absorbance, fluorescence, and scattered light were measured as parameters for biological samples validating a clear distinction between individual populations, which were not separable by a single parameter. Microbial growth kinetics and growth inhibition assays were realized with the developed setup. Compared to traditional approaches, the fiber embedded optofluidic construct provides flexibility, simplicity, cost reduction, and improved signal sensitivity.

An optofluidic setup, as demonstrated here, can be transformed for enhancing detection methods in various microfluidic applications.^5,12^ With future improvements based on the same concept and technology, multiple fibers or fiber bundles can be incorporated for enhancing multi-parameter measurements. Similarly, optical fibers can be coupled with waveguides, air-mirrors, and filters in order to extend the range of applications like analyte detection,^24,37^ disease marker detection,^61^ single cell analysis,^25^ chemical analysis,^3,62^ and cytometry.^20,55^ The developed optofluidics setup holds promise for developing an all-in-one multi-parametric detection system for various biochemical, microbial, and other relevant applications.

## SUPPLEMENTARY MATERIAL

See the supplementary material for LSM data, droplet signal patterns, and the growth curve of Streptomyces.
